# Platelet optimization in high-risk ALBI grade 3 patients undergoing invasive procedures: real-world evidence from lusutrombopag therapy

**DOI:** 10.3389/fphar.2026.1817055

**Published:** 2026-07-09

**Authors:** Kongcai Zhu, Fang Xiong, Haihong Bai, Wei Liu

**Affiliations:** 1 Department of Pharmacy, Beijing Youan Hospital, Capital Medical University, Beijing, China; 2 Department of Oncology, Beijing Youan Hospital, Capital Medical University, Beijing, China

**Keywords:** ALBI grade, hepatocellular carcinoma, lusutrombopag, peri-procedural management, thrombocytopenia

## Abstract

**Background:**

Evidence regarding lusutrombopag use in patients with hepatocellular carcinoma (HCC) and poor hepatic reserve remains limited, particularly in ALBI grade 3 patients. This study aimed to explore peri-procedural platelet outcomes after lusutrombopag treatment in HCC patients stratified by ALBI grade, with particular attention to the ALBI grade 3 subgroup.

**Methods:**

We conducted a single-center retrospective study of 76 patients with HCC and thrombocytopenia who received lusutrombopag before planned procedures. Patients were stratified as ALBI grades 1–2 or ALBI grade 3. The primary endpoint was peri-procedural platelet count, defined as the platelet on the procedure day or the closest available value within a ±2-day window. Exploratory analyses included an adjusted general linear model, logistic regression for non-response, and per-protocol sensitivity analyses excluding patients who received rescue therapy.

**Results:**

Among 76 patients, 58 had ALBI grades 1–2 and 18 had ALBI grade 3. The primary endpoint was evaluable in 60 patients, including 46 with ALBI grades 1–2 and 14 with ALBI grade 3. Median peri-procedural platelet counts were 59.0 (50.0–69.0) × 10^9^/L in the ALBI grades 1–2 group and 69.0 (41.8–82.8) × 10^9^/L in the ALBI grade 3 group (*p = 0.345*). Responder rates were 84.5% (49/58) and 77.8% (14/18), respectively (*p = 0.493*). In adjusted analyses, ALBI grade 3 was not independently associated with lower peri-procedural platelet count or non-response after accounting for baseline platelet count and relevant covariates. Per-protocol sensitivity analyses yielded directionally similar findings. No bleeding or thrombotic events were observed in the ALBI grade 3 group, although safety analyses were descriptive.

**Conclusion:**

In this selected real-world HCC cohort, ALBI grade 3 was not independently associated with lower peri-procedural platelet count after lusutrombopag treatment. These findings are exploratory and should not be interpreted as evidence of equivalent efficacy or established safety across ALBI strata. Larger prospective studies are needed to validate these results.

## 1 Introduction

Hepatocellular carcinoma (HCC) remains a leading cause of cancer-related mortality worldwide (Sung et al., 2021). Many patients with HCC have underlying chronic liver disease or cirrhosis, in which thrombocytopenia is common and multifactorial, involving portal hypertension-related splenic sequestration and reduced hepatic production of thrombopoietin (Zhao et al., 2024; Mitchell et al., 2016; Desai and Subramanian, 2021; Martin et al., 1997). Severe thrombocytopenia (platelet count <50 × 10^9^/L) poses a major obstacle to invasive procedures, including transarterial chemoembolization and radiofrequency ablation (RFA), due to the increased risk of peri-procedural bleeding (Scharf, 2021; Wang et al., 2019; Zhou et al., 2025).

Platelet transfusion has traditionally been used to mitigate bleeding risk; however, it is associated with transient efficacy, logistical constraints, and potential complications such as transfusion reactions and infectious transmission (Garraud et al., 2023). Lusutrombopag, an orally administered thrombopoietin receptor agonist (TPO-RA), was developed as a pharmacologic alternative to platelet transfusion. The efficacy and safety of lusutrombopag were established in the L-PLUS 1 and L-PLUS 2 phase III trials, which demonstrated a significant reduction in the need for rescue platelet transfusion in patients with CLD undergoing elective procedures (Hidaka et al., 2019; Peck-Radosavljevic et al., 2019). Subsequent real-world studies and post-marketing surveillance analyses have largely confirmed these findings in routine clinical practice (Nomoto et al., 2020; Gallo et al., 2024; Sasaki et al., 2019; Yoshiji et al., 2023).

Despite accumulating evidence supporting the use of lusutrombopag, several clinically relevant questions remain incompletely addressed. Previous studies have evaluated platelet response using outcomes such as avoidance of platelet transfusion, achievement of platelet counts ≥50 × 10^9^/L, and changes in platelet counts after treatment. In addition, hepatic reserve has been assessed mainly using Child–Pugh classification or Albumin-Bilirubin (ALBI) score as a continuous predictor variable. Previous work has suggested that baseline ALBI score is not independently associated with platelet count increase following lusutrombopag treatment (Wada et al., 2021). However, limited data are available regarding peri-procedural platelet responses in HCC patients stratified by ALBI grade, particularly those classified as ALBI grade 3.

ALBI grade is an objective liver function assessment tool developed and widely used in patients with HCC, calculated solely from serum albumin and bilirubin levels (Johnson et al., 2015; Zhao et al., 2020; Demirtas et al., 2021). Although patients with ALBI grade 3 generally exhibit poorer hepatic reserve than those with ALBI grades 1–2, ALBI grade 3 should not be considered equivalent to Child-Pugh class C. Therefore, ALBI-based stratification may provide complementary information for evaluating peri-procedural platelet response in HCC patients, but its relevance in lusutrombopag-treated patients remains unclear.

Therefore, this study aimed to describe peri-procedural platelet response and safety outcomes following lusutrombopag treatment in HCC patients stratified according to ALBI grade, with particular attention to the ALBI grade 3 subgroup. In addition, we sought to explore clinical factors associated with peri-procedural platelet response in a real-world setting.

## 2 Methods

### 2.1 Study design and population

**FIGURE 1:**
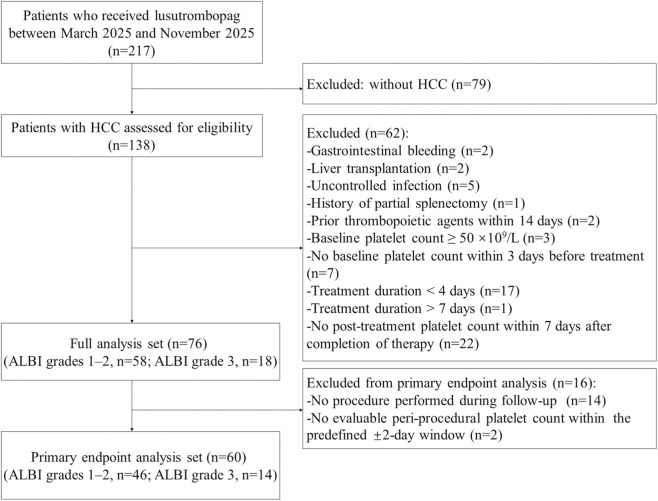
Patient selection flow diagram. The flowchart summarizes the patient screening process, exclusion criteria, and analysis sets. Abbreviations: ALBI, albumin-bilirubin; HCC, hepatocellular carcinoma.

This retrospective single-center study was conducted at Beijing Youan Hospital from March to November 2025. Consecutive patients with HCC who received lusutrombopag for planned invasive procedures were screened for eligibility. The patient selection process is shown in Figure 1.

The major inclusion criteria were: age ≥18 years, confirmed diagnosis of HCC, baseline thrombocytopenia (platelet count <50 × 10^9^/L), availability of laboratory data required for ALBI grade calculation. Patients were excluded if they: received platelet transfusion or thrombopoietic agents within 14 days prior to treatment, had active infection or uncontrolled bleeding, received lusutrombopag for fewer than 4 days or more than 7 days, had no baseline platelet measurement within 3 days before lusutrombopag initiation, had no post-treatment platelet measurement within 7 days after treatment completion, or had a history of splenectomy or liver transplantation. Patients receiving lusutrombopag for fewer than 4 days or more than 7 days were excluded to reduce heterogeneity related to markedly insufficient or non-standard drug exposure.

Eligible patients were stratified by ALBI grade as ALBI grades 1–2 or ALBI grade 3. ALBI grade was calculated using serum albumin and total bilirubin. Child–Pugh score and class were retrospectively assessed from available clinical and laboratory records and summarized descriptively, but were not used as the primary stratification variable.

All patients received lusutrombopag at a fixed dose of 3 mg once daily. Treatment duration (4–7 days) and timing of the procedure were determined by the treating physician based on platelet response and procedural scheduling in routine clinical practice. Patients requiring rescue therapy, defined as platelet transfusion or recombinant human thrombopoietin (rhTPO), during the treatment or peri-procedural period were included in the full analysis set (FAS) but excluded from the per-protocol (PP) sensitivity analysis.

### 2.2 Analysis sets

The full analysis set included all eligible patients. The primary endpoint analysis set included patients with an available peri-procedural platelet count, defined as a platelet measurement obtained on the day of procedure or the closest available value within ±2 days. When multiple platelet measurements were available, the value obtained on the procedure day was prioritized, otherwise, the value closest to the procedure day was used. Patients without platelet measurements within this peri-procedural window were excluded from the primary endpoint analysis. The per-protocol sensitivity set excluded patients who received rescue therapy during the treatment or peri-procedural period. For safety analyses, all eligible patients were assessed descriptively, and patients categorized as “non-procedure” were excluded from procedure-related safety analyses.

### 2.3 Endpoints

The primary endpoint was the peri-procedural platelet count, defined as the measurement obtained on the day of procedure or the closest available value within ±2 days. Secondary endpoints included the responder rate, defined as the proportion of patients achieving a platelet count ≥50 × 10^9^/L with an absolute increase of ≥20 × 10^9^/L from baseline. Safety outcomes included clinically significant bleeding events during the peri-procedural period and thrombotic events. Thrombotic events were defined as clinically documented events confirmed by imaging or physician diagnosis during routine follow-up.

### 2.4 Data collection and laboratory measurements

Clinical and laboratory data were extracted from the institutional electronic medical record system. Data collected included demographic characteristics, procedure type, lusutrombopag treatment duration, platelet measurements, liver function parameters, portal vein diameter, rescue therapy, bleeding events, thrombotic events, and concomitant factors potentially affecting platelet count or bleeding risk, including antiplatelet agents, anticoagulants, portal vein thrombosis, and splenectomy history. Concomitant medications and clinical conditions were identified through review of medication records, physician notes, imaging reports, and discharge summaries. Owing to the limited sample size and small number of outcome events, these variables were summarized descriptively and were not all included in multivariable models.

Baseline platelet counts were obtained within 3 days before the first lusutrombopag dose. At least one post-treatment platelet measurement within 7 days after treatment completion was required for eligibility and responder assessment. For the primary endpoint, the procedure-day platelet count was prioritized; if unavailable, the closest value within the predefined ±2-day window was selected. Peripheral venous blood samples were collected in K2-EDTA anticoagulant tubes and analyzed within 4 h of collection without additional sample processing. Platelet counts were measured using the sheath flow impedance method (PLT-I) on a Sysmex XN automated hematology analyzer in the institutional central laboratory. Because blood sampling was performed as part of routine clinical care, the exact clock-time intervals between blood collection, daily lusutrombopag administration, and the procedure were not consistently available.

### 2.5 Statistical analysis

Continuous variables were expressed as median with interquartile range (IQR) unless otherwise specified. Categorical variables were expressed as number and percentage. Comparisons between groups were performed using the Mann–Whitney U test for continuous variables, and the χ^2^ test or Fisher’s exact test for categorical variables.

Spearman correlation analyses were conducted as exploratory assessments to evaluate potential associations between platelet response and continuous clinical variables. An adjusted analysis of the primary endpoint was performed using a general linear model (GLM). Peri-procedural platelet count was entered as the dependent variable, ALBI grade group as a fixed factor, with ALBI grades 1–2 as the reference category, and baseline platelet count and portal vein diameter as prespecified covariates. Baseline platelet count was selected because it is a well-recognized determinant of platelet response following TPO-RA therapy, whereas portal vein diameter was included as a surrogate marker of portal hypertension and splenic sequestration. Model assumptions were assessed by examination of residual distributions, residual-versus-fitted plots, and Levene’s test for homogeneity of variance.

Exploratory logistic regression was performed with non-response as the dependent variable for the secondary endpoint, as an exploratory analysis to evaluate factors associated with failure to achieve platelet response. Given the limited number of non-response events, the multivariable model was restricted to prespecified clinically relevant covariates (ALBI grade group and baseline platelet count) to minimize overfitting. To evaluate the robustness of the findings, a per-protocol (PP) sensitivity analysis was performed by excluding patients who received rescue therapy. For PP sensitivity analyses, endpoint-specific evaluable denominators were used because the availability of peri-procedural platelet measurements and responder status differed across patients. Effect estimates were reported with 95% confidence intervals where applicable.

Missing data were not imputed. Patients without peri-procedural platelet measurements within the predefined ±2-day window were excluded from the primary endpoint analysis but retained in descriptive baseline summaries and other analyses when relevant data were available.

No formal sample size calculation was performed because this was a retrospective exploratory study based on available real-world cases. The ALBI grade 3 subgroup was small; therefore, the study was underpowered for definitive between-group comparisons and at risk of type II error. All comparative analyses should be interpreted as exploratory and hypothesis-generating. All statistical tests were two-sided, and *p < 0.05* was considered statistically significant. All analyses were performed using SPSS version 26.0 (IBM Corp., Armonk, NY, USA).

## 3 Results

### 3.1 Baseline characteristics

A total of 76 patients with HCC were included in the full analysis set, comprising 58 patients with ALBI grades 1–2 and 18 patients with ALBI grade 3. Baseline characteristics are summarized in Table 1. Patients with ALBI grade 3 exhibited features consistent with poorer hepatic reserve, including lower serum albumin levels and higher bilirubin concentrations. Among the 18 patients with ALBI grade 3, 7 were classified as Child–Pugh class C and 11 as Child–Pugh class B. Concomitant factors potentially affecting platelet count or bleeding risk were systematically reviewed. No patient was receiving antiplatelet or anticoagulant therapy at baseline, and prior splenectomy was an exclusion criterion. Pre-existing portal vein thrombosis was identified in 10 patients overall, including 6 patients in the ALBI grades 1–2 group and 4 patients in the ALBI grade 3 group. Baseline platelet counts was numerically lower in the ALBI grades 1–2 group than in the ALBI grade 3 group, whereas portal vein diameter was similar between groups.

**TABLE 1:** Baseline demographic and clinical characteristics of the study population.

Characteristic	ALBI grades 1–2 (n = 58)	ALBI grade 3 (n = 18)	*p-*value
Age, years	58.5 (51.8–67.0)	61.0 (57.3–68.0)	*0.210*
Male sex, n (%)	44 (75.9%)	18 (100.0%)	*0.032*
BMI, kg/m^2^	23.3 (20.7–25.4)	23.4 (21.8–26.1)	*0.700*
Etiology, n (%)	​	​	*0.509*
HBV	41 (70.7%)	12 (66.7%)	​
HCV	4 (6.9%)	0 (0.0%)	​
Others	13 (22.4%)	6 (33.3%)	​
Baseline PLT, × 10^9^/L	38.0 (32.0–44.3)	42.5 (32.8–47.0)	*0.208*
Albumin, g/L	33.3 (31.0–37.0)	27.1 (25.3–28.4)	*<0.001*
Total bilirubin, μmol/L	29.6 (22.5–42.9)	43.0 (33.7–47.2)	*0.003*
PT-INR	1.21 (1.13–1.29)	1.31 (1.20–1.46)	*0.011*
Portal vein diameter, mm	14.0 (12.0–17.0)	14.0 (11.0–17.0)	*0.814*
Creatinine, μmol/L	61.0 (52.0–70.0)	73.0 (64.8–86.0)	*0.004*
Hemoglobin, g/L	113.0 (97.8–123.8)	100.0 (84.3–123.5)	*0.219*
Child-Pugh score	7.0 (6.0–7.5)	9.0 (8.0–10.0)	*<0.001*
Child–Pugh class, n (%)	​	​	*<0.001*
A	21 (36.2%)	0 (0.0%)	​
B	37 (63.8%)	11 (61.1%)	​
C	0 (0.0%)	7 (38.9%)	​
Pre-existing portal vein thrombosis	6 (10.3%)	4 (22.2%)	*0.235*
Procedure type, n (%)	​	​	*0.708*
RFA	35 (60.3%)	13 (72.2%)	​
Transarterial lipiodol embolization	5 (8.6%)	1 (5.6%)	​
Hepatectomy	2 (3.4%)	0 (0.0%)	​
Others	6 (10.3%)	0 (0.0%)	​
No procedure	10 (17.2%)	4 (22.2%)	​

Data are presented as median (interquartile range) or n (%). P-values were calculated using the Mann–Whitney U test for continuous variables and the χ^2^ test or Fisher’s exact test for categorical variables. Abbreviations: ALBI, albumin–bilirubin; BMI, body mass index; HBV, hepatitis B virus; HCV, hepatitis C virus; PLT, platelet count; PT-INR, prothrombin time–international normalized ratio; RFA, radiofrequency ablation.

### 3.2 Peri-procedural platelet response

**FIGURE 2:**
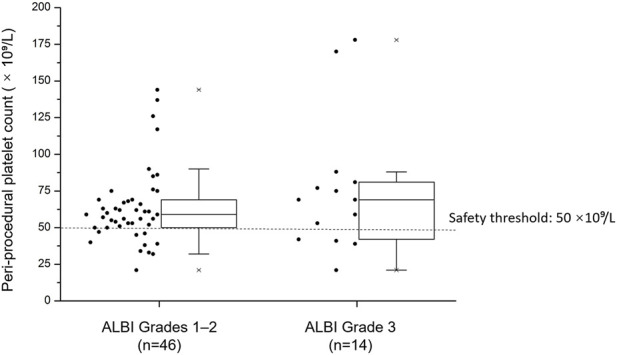
Peri-procedural platelet counts by ALBI grade. Legends: Distribution of peri-procedural platelet counts in HCC patients treated with lusutrombopag, stratified by ALBI grade (grades 1–2 vs. grade 3). Peri-procedural platelet count was defined as the measurement obtained on the day of procedure or the closest available value within ±2 days. Box plots represent median values, interquartile ranges (IQR), and whiskers (1.5 × IQR). The horizontal dashed line indicates the commonly applied procedural safety threshold (50 × 10^9^/L). Each black dot represents an individual patient, while “ × ” symbols denote outliers. Abbreviation: ALBI, albumin–bilirubin.

Of the 76 patients included in the full analysis set, 60 had evaluable peri-procedural platelet measurements within the predefined ±2-day assessment window and were included in the primary endpoint analysis, including 46 patients with ALBI grades 1–2 and 14 patients with ALBI grade 3. Platelet counts were measured on the day of procedure in 13/46 patients with ALBI grades 1–2 and 3/14 patients with ALBI grade 3; the remaining measurements were obtained within the predefined ±2-day window (Table 2). The median peri-procedural platelet count was 59.0 × 10^9^/L (IQR 50.0–69.0) in patients with ALBI grades 1–2 and 69 × 10^9^/L (IQR 41.8–82.8) in patients with ALBI grade 3 *(p = 0.345)*. Platelet distributions are illustrated in Figure 2.

**TABLE 2:** Primary peri-procedural platelet outcome and timing of platelet measurement.

Variable	ALBI grades 1–2 (n = 46)	ALBI grade 3 (n = 14)	*p*-value
Peri-procedural PLT, × 10^9^/L	59.0 (50.0–69.0)	69.0 (41.8–82.8)	*0.345*
Timing of selected platelet measurement			*0.716*
Day −2, n (%)	5 (10.9%)	0 (0.0%)	​
Day −1, n (%)	13 (28.3%)	5 (35.7%)	​
Day 0, n (%)	13 (28.3%)	3 (21.4%)	​
Day +1, n (%)	14 (30.4%)	6 (42.9%)	​
Day +2, n (%)	1 (2.2%)	0 (0.0%)	​

Values are presented as median (interquartile range) or n (%). The primary endpoint analysis included patients with platelet measurements obtained on the day of procedure or within the predefined ±2-day assessment window. Day 0 indicates the day of procedure. The p-value for timing of platelet measurement refers to the comparison of measurement timing distribution between groups. Abbreviations: ALBI, albumin–bilirubin; PLT, platelet count.

In exploratory correlation analyses, peri-procedural platelet count was positively associated with baseline platelet count (Spearman ρ = 0.37, *p = 0.004*) and portal vein diameter (Spearman ρ = 0.48, *p < 0.001*), whereas no meaningful association was observed with baseline ALBI score (Spearman ρ = 0.03, *p = 0.823*).

Results of the adjusted general linear model are presented in Table 3. In the adjusted general linear model, no independent association was detected between ALBI grade 3 and peri-procedural platelet count (β = −10.57, 95% CI -29.33 to 8.19; *p = 0.263*). Baseline platelet count (β = 1.04, 95% CI 0.10 to 1.99; *p = 0.031*) and portal vein diameter (β = 3.56, 95% CI 1.34 to 5.78; *p = 0.002*) were associated with higher peri-procedural platelet counts. Levene’s test did not indicate significant heterogeneity of variance (*p = 0.085*), although residual normality was imperfect; therefore, the adjusted model was interpreted cautiously as an exploratory analysis.

**TABLE 3:** Adjusted general linear model for peri-procedural platelet count.

Variable	β coefficient (SE)	95% CI	*p-*value
ALBI grade 3	−10.57 (9.34)	−29.33–8.19	*0.263*
Baseline PLT	1.04 (0.47)	0.10–1.99	*0.031*
Portal vein diameter	3.56 (1.11)	1.34–5.78	*0.002*

β coefficients were estimated using an adjusted general linear model with peri-procedural platelet count as the dependent variable. ALBI, grade group was specified as a fixed factor, with ALBI, grades 1–2 as the reference category. Baseline platelet count and portal vein diameter were included as covariates. For continuous covariates, β coefficients represent the adjusted change in peri-procedural platelet count per 1 × 10^9^/L increase in baseline platelet count or per 1-mm increase in portal vein diameter. Results are expressed as β coefficient (standard error) with 95% confidence intervals. Abbreviations: ALBI, albumin–bilirubin; PLT, platelet count; SE: standard error; CI: confidence interval.

### 3.3 Responder analysis

In the full analysis set, responder rates were 84.5% (49/58) in ALBI grades 1%–2% and 77.8% (14/18) in ALBI grade 3 *(p = 0.493)*. In the exploratory logistic regression with non-response as the dependent variable, ALBI grade 3 was not independently associated with non-response after adjustment for baseline platelet count (adjusted OR 1.75, 95% CI 0.45–6.75, *p = 0.416*). Pre-existing portal vein thrombosis was evaluated in univariate analysis and was not significantly associated with non-response (OR 2.40, 95% CI 0.53–10.87; *p = 0.256*). The wide confidence interval indicated substantial statistical uncertainty, and this analysis should be interpreted as exploratory. Results are presented in Table 4.

**TABLE 4:** Exploratory logistic regression analysis for non-response.

Candidate variable	Univariate OR (95% CI)	Univariate *p*-value	Multivariable* OR (95% CI)	Multivariable* *p*-value
ALBI grade 3 (ref: ALBI grades 1–2)	1.56 (0.42–5.82)	0.512	1.75 (0.45–6.75)	0.416
Baseline PLT	1.03 (0.96–1.11)	0.397	1.04 (0.97–1.11)	0.335
Portal vein diameter	1.03 (0.86–1.23)	0.770		
Age	1.03 (0.97–1.09)	0.357		
Male sex (ref: female)	0.32 (0.04–2.70)	0.295		
BMI	1.15 (0.94–1.40)	0.183		
PT-INR	0.14 (0.003–5.78)	0.298		
Creatinine	0.96 (0.89–1.03)	0.234		
Hemoglobin	0.98 (0.74–1.30)	0.885		
Pre-existing portal vein thrombosis	2.40 (0.53–10.87)	0.256		

Odds ratios (ORs) were estimated using univariate binary logistic regression with non-response as the dependent variable. Univariate analyses were exploratory and were not used for automatic variable selection. *Variables considered clinically relevant (ALBI, grade and baseline platelet count) were entered into the multivariable model irrespective of univariate significance. Wide confidence intervals reflect the limited number of non-response events. Abbreviations: ALBI, albumin–bilirubin; PLT, platelet count; OR: odds ratio; CI: confidence interval; BMI: body mass index; PT-INR: prothrombin time-international normalized ratio.

### 3.4 Per-protocol sensitivity analysis

Per-protocol (PP) sensitivity analyses were performed after excluding patients who received platelet transfusion or recombinant human thrombopoietin during the treatment or peri-procedural period. Because the availability of evaluable data differed between the peri-procedural platelet endpoint and the responder endpoint, denominators are reported separately for each analysis. Results of the PP sensitivity analyses are presented in Table 5.

**TABLE 5:** Per-protocol sensitivity analyses after exclusion of rescue therapy.

Outcome	ALBI grades 1–2	ALBI grade 3	*p*-value
Peri-procedural PLT, × 10^9^/L	59.0 (50.3–73.5); N = 32	69.0 (47.0–84.5); N = 13	*0.270*
Responder rate, n/N (%)	35/42 (83.3%)	13/17 (76.5%)	*0.713*

Values are presented as median (interquartile range) or n/N (%). Per-protocol analyses excluded patients who received platelet transfusion or recombinant human thrombopoietin during the treatment or peri-procedural period. Denominators differed between endpoints because the peri-procedural platelet endpoint required an evaluable platelet count within the predefined ±2-day window, whereas the responder endpoint was evaluated based on available post-treatment platelet response data. Abbreviations: ALBI, albumin-bilirubin; PLT, platelet count.

For the peri-procedural platelet endpoint, the PP-evaluable population included 32 patients with ALBI grades 1–2 and 13 patients with ALBI grade 3. Median peri-procedural platelet counts were 59.0 × 10^9^/L (IQR 50.3–73.5) in patients with ALBI grades 1–2 and 69.0 × 10^9^/L (IQR 47.0–84.5) in patients with ALBI grade 3 (*p = 0.270*). For the responder endpoint, responder rates were 83.3% (35/42) and 76.5%, respectively (*p = 0.713*). These findings were directionally similar to the primary analyses but should be interpreted descriptively because of the reduced sample size, varying endpoint-specific denominators, and non-random use of rescue therapy.

### 3.5 Real-world dosing patterns

Treatment duration ranged from 4 to 7 days in the included cohort. Overall, 24 patients (31.6%) received a short-course regimen of lusutrombopag for 4–6 days, whereas 52 patients (68.4%) received the standard 7-day regimen. Short-course treatment was used in 15 of 58 patients (25.9%) with ALBI grades 1–2 and in 9 of 18 patients (50.0%) with ALBI grade 3. Platelet outcomes according to treatment duration were assessed descriptively. Among patients with evaluable peri-procedural platelet measurements, the median peri-procedural platelet count was 61.0 × 10^9^/L (IQR 50.0–81.0) in the short-course group (n = 19) and 59.0 × 10^9^/L (IQR 46.0–75.0) in the 7-day group (n = 41). In the full analysis set, responder rates were 87.5% (21/24) and 80.8% (42/52), respectively. Because treatment duration was determined by treating physicians according to platelet monitoring and procedural scheduling rather than by a predefined protocol, these findings should be interpreted descriptively and not as evidence of comparative efficacy between dosing patterns.

### 3.6 Safety outcomes

Safety outcomes were assessed descriptively because of the limited number of events (Table 6). Procedure-related bleeding events were summarized among patients who underwent an invasive procedure, whereas thrombotic events were assessed in the full analysis set during routine follow-up.

**TABLE 6:** Descriptive safety outcomes by ALBI grade.

Outcome	Analysis population	ALBI grades 1–2	ALBI grade 3
Thrombotic events, n/N (%)	Full analysis set	1/58 (1.7%)	0/18 (0.0%)
Any bleeding event, n/N (%)	Full analysis set	4/58 (6.9%)	0/18 (0.0%)
Procedure-related bleeding, n/N (%)	Patients undergoing invasive procedures	3/48 (6.3%)	0/14 (0.0%)
Fatal bleeding, n/N (%)	Full analysis set	0/58 (0.0%)	0/18 (0.0%)
Treatment-related death, n/N (%)	Full analysis set	0/58 (0.0%)	0/18 (0.0%)

Safety outcomes were summarized descriptively. Thrombotic events, any bleeding event, fatal bleeding, and treatment-related death were evaluated in the full analysis set. Procedure-related bleeding was evaluated among patients who underwent invasive procedures. One bleeding event occurred in a patient without an invasive procedure and was included in any bleeding event but not classified as procedure-related bleeding. No formal between-group statistical comparison was performed because of the limited number of events. Abbreviation: ALBI, albumin-bilirubin.

In the full analysis set, one thrombotic event was observed in the ALBI grades 1–2 group, consisting of portal vein thrombosis diagnosed during follow-up in a patient who underwent ablation for HCC. No thrombotic events were observed in the ALBI grade 3 group.

Bleeding events were observed in 4 patients in the ALBI grades 1–2 group and in no patients in the ALBI grade 3 group. Three bleeding events were procedure-related, occurring after pulmonary ablation, transarterial lipiodol embolization, and hepatic resection, respectively. The remaining bleeding event occurred in a patient without an invasive procedure during follow-up and was therefore not classified as procedure-related bleeding. No fatal bleeding or treatment-related death was documented. No formal between-group statistical comparison was performed because of the limited number of events.

## 4 Discussion

### 4.1 Main findings

In this retrospective exploratory real-world study, we evaluated peri-procedural platelet responses and safety outcomes after lusutrombopag treatment in patients with HCC stratified by ALBI grade, with particular attention to the ALBI grade 3 subgroup. Peri-procedural platelet responses were observed across ALBI strata, and no statistically significant difference in peri-procedural platelet count was detected between patients with ALBI grades 1–2 and those with ALBI grade 3. In exploratory adjusted analyses, ALBI grade 3 was not independently associated with lower peri-procedural platelet count after adjustment for baseline platelet count and portal vein diameter. However, because of the retrospective design, small ALBI grade 3 subgroup, and heterogeneous real-world management, these findings should be interpreted as hypothesis-generating rather than definitive.

### 4.2 Rationale for ALBI-based stratification

The choice of ALBI grade as the primary stratification variable was based on the HCC-specific context of the present study. ALBI grade was originally developed and validated in patients with HCC as a simple, objective, evidence-based method for assessing liver function, using only serum albumin and total bilirubin (Johnson et al., 2015). Subsequent multicenter validation further supported the prognostic relevance of ALBI across BCLC stages and major HCC treatment settings. For example, Pinato et al. evaluated 2,426 patients with HCC from Europe, the United States, and Asia and found that ALBI grade was significantly associated with overall survival after surgical resection, transarterial treatment, and sorafenib therapy, and retained independent prognostic value across BCLC stages and geographic cohorts (Pinato et al., 2017). This HCC-specific evidence, together with the fully laboratory-based nature of ALBI, made it suitable for the present retrospective real-world analysis, in which subjective Child–Pugh components such as ascites and hepatic encephalopathy may be inconsistently documented or graded.

Nevertheless, ALBI grade and Child–Pugh class capture overlapping but non-identical dimensions of liver function. Child–Pugh classification incorporates synthetic function and clinical decompensation, including prothrombin time, ascites, and encephalopathy, whereas ALBI reflects an albumin–bilirubin-defined dimension of hepatic reserve. For this reason, Child–Pugh score and class were retrospectively assessed and reported descriptively. In the present cohort, 7 of 18 patients with ALBI grade 3 were Child–Pugh class C, whereas the remaining patients were Child–Pugh class B. This distribution demonstrates that ALBI grade 3 should not be implicitly equated with Child–Pugh class C or with severe hepatic impairment as defined by Child–Pugh criteria; therefore, the present findings should be interpreted within an ALBI-defined HCC subgroup rather than extrapolated directly to all Child–Pugh class C patients.

### 4.3 Comparison with previous lusutrombopag studies and novelty of the present study

Previous pivotal trials established lusutrombopag as an effective option for increasing platelet counts and reducing the need for platelet transfusion in patients with chronic liver disease and thrombocytopenia undergoing planned invasive procedures (Hidaka et al., 2019; Peck-Radosavljevic et al., 2019). In L-PLUS 1 and L-PLUS 2, lusutrombopag significantly reduced the proportion of patients requiring platelet transfusion compared with placebo, and platelet counts generally reached clinically relevant procedural thresholds within several days after treatment initiation (Hidaka et al., 2019; Peck-Radosavljevic et al., 2019). Real-world evidence has further supported the effectiveness and safety of lusutrombopag in routine clinical practice. In a Japanese post-marketing surveillance study, 282 of 300 patients undergoing invasive procedures avoided preoperative platelet transfusion, and the mean maximum platelet count increase was 41.7 × 10^9^/L (Sasaki et al., 2019). However, Child–Pugh class C patients were excluded from the effectiveness analysis because this was outside the approved indication in Japan. More recently, the European REALITY study reported that lusutrombopag increased platelet counts from a median of 37 × 10^9^/L to 58 × 10^9^/L, with 84% of treatment cycles avoiding platelet transfusion (Gallo et al., 2024). These studies support the efficacy of lusutrombopag for peri-procedural platelet management, but they were not designed to evaluate HCC patients stratified by ALBI grade or to specifically characterize ALBI grade 3 patients.

Previous studies have also explored predictors of response to lusutrombopag. Wada et al. reported that baseline ALBI score was not significantly associated with platelet count change, whereas anti-GPIIb/IIIa antibody-producing B cells were independently associated with platelet count increase (Wada et al., 2021). However, that study was conducted in a mixed chronic liver disease population, only half of whom had liver cancer, and it evaluated maximum platelet count changes during a post-treatment window rather than peri-procedural platelet counts according to ALBI grade. Therefore, the present study does not claim that ALBI is a novel predictor of lusutrombopag response. Instead, its contribution is to provide exploratory, ALBI-stratified, HCC-specific real-world data, with explicit attention to the ALBI grade 3 subgroup and actual procedure-window platelet measurements.

The study by Flamm et al. is particularly relevant because it directly evaluated lusutrombopag in Child–Pugh class C patients using data from a phase 1/2 Child–Pugh class C study, L-PLUS 2, and post-marketing surveillance (Flamm et al., 2022). That analysis included a limited number of Child–Pugh class C patients, including 5 patients from a phase 1/2 study, 3 from L-PLUS 2, and 27 from post-marketing surveillance. From an efficacy or treatment-effect perspective, among lusutrombopag-treated Child–Pugh class C patients who did not receive platelet transfusion, the reported median maximum platelet counts were 88.5 × 10^9^/L in the phase 1/2 study, 80 × 10^9^/L in L-PLUS 2, and 91 × 10^9^/L in post-marketing surveillance. From a safety perspective, no treatment-related adverse events or treatment-related serious adverse events were observed, and one non-serious portal vein thrombosis event in the phase 1/2 study was not considered treatment-related. These findings suggest that lusutrombopag can increase platelet counts in Child–Pugh class C patients and may be tolerated in this population, although the limited sample size requires cautious interpretation.

The present study differs from and complements Flamm et al. in two main respects. First, the present study focused on HCC patients stratified by ALBI grade. In our cohort, only 7 of 18 patients with ALBI grade 3 were Child–Pugh class C, confirming that ALBI grade 3 and Child–Pugh class C are overlapping but non-identical categories. Second, Flamm et al. primarily addressed pharmacokinetic exposure, maximum platelet response, and safety in Child–Pugh class C patients, whereas our study evaluated peri-procedural platelet count within an actual procedure-related assessment window in an ALBI-defined HCC cohort. Accordingly, the present study should not be viewed as demonstrating equivalence across ALBI strata or duplicating existing Child–Pugh class C evidence. Rather, it provides complementary, hypothesis-generating real-world data for an ALBI-defined HCC subgroup that has been insufficiently characterized in previous studies.

### 4.4 Pharmacokinetic considerations

Previous pharmacokinetic analyses have reported that patients with advanced hepatic impairment (Child–Pugh class C) exhibit approximately 20% reductions in total plasma Cmax and AUC of lusutrombopag compared with those with milder hepatic dysfunction (Flamm et al., 2022). Although reduced total systemic exposure might be expected to attenuate platelet response, ALBI grade 3 was not independently associated with lower peri-procedural platelet count in the present adjusted analysis. In the context of prior Child–Pugh class C pharmacokinetic data, this observation is compatible with the possibility that lower total exposure may not preclude a platelet response. One possible hypothesis is that, because lusutrombopag is highly protein-bound, lower albumin levels may alter the relationship between total and unbound drug concentrations (U.S. Food and Drug Administration, 2018; Benet and Hoener, 2002; Ulldemolins et al., 2011; Łuszczyńska et al., 2019). However, this explanation remains speculative. The present study did not measure total or unbound lusutrombopag concentrations, and ALBI grade 3 should not be considered equivalent to Child–Pugh class C. Therefore, these pharmacokinetic considerations should be regarded as hypothesis-generating only. Prospective pharmacokinetic–pharmacodynamics studies incorporating direct measurement of unbound lusutrombopag concentrations are required to clarify whether altered protein binding contributes to platelet response in patients with reduced hepatic reserve.

### 4.5 Determinants of platelet response

Platelet response to lusutrombopag is likely multifactorial and cannot be explained solely by hepatic reserve classification. Thrombocytopenia in chronic liver disease reflects several interacting mechanisms, including portal hypertension-related splenic sequestration, reduced thrombopoietin production, bone marrow suppression, immune-mediated platelet destruction, and disease severity (Mitchell et al., 2016; Martin et al., 1997). In addition to these baseline mechanisms, the observed peri-procedural platelet count after lusutrombopag may be influenced by baseline platelet count, spleen-related platelet sequestration, inflammatory or immune factors, rescue therapy, treatment duration, and timing of platelet measurement relative to the procedure.

Consistent with this multifactorial framework, Wada et al. reported that baseline ALBI score was not significantly associated with platelet count increase, whereas anti-GPIIb/IIIa antibody-producing B cells and spleen size were associated with platelet count changes (Wada et al., 2021). The present study is consistent with this concept, but it should not be interpreted as establishing ALBI grade as a predictor or non-predictor of lusutrombopag response. Rather, it indicates that, in this selected real-world HCC cohort, ALBI grade 3 was not independently associated with lower peri-procedural platelet count in exploratory adjusted analysis.

The baseline platelet distribution in this cohort also requires cautious interpretation. In chronic liver disease populations, poorer hepatic reserve may be accompanied by lower platelet counts. However, this expected relationship was not observed in the present cohort. Baseline platelet count was numerically lower in patients with ALBI grades 1–2 than in those with ALBI grade 3, with median values of 38.0 × 10^9^/L and 42.5 × 10^9^/L, respectively. This apparent imbalance should be interpreted in the context of the study design. Patients were not enrolled as an unselected HCC population; they were selected for lusutrombopag treatment before planned procedures. Thus, patients with better hepatic reserve may have proceeded to invasive procedures despite lower baseline platelet counts, whereas patients with poorer hepatic reserve may have been selected for procedures only when platelet counts were relatively higher or clinically manageable.

This imbalance is relevant because baseline platelet count was positively associated with peri-procedural platelet count in the adjusted general linear model. The slightly higher baseline platelet count in the ALBI grade 3 group may therefore have contributed to the absence of a lower peri-procedural platelet count in this subgroup. For this reason, baseline platelet count was included as a covariate in the adjusted model. After adjustment for baseline platelet count and portal vein diameter, ALBI grade 3 was not independently associated with lower peri-procedural platelet count. However, because of the small sample size and retrospective non-randomized design, this adjustment should be considered exploratory and cannot exclude residual confounding.

Portal vein diameter was also positively associated with peri-procedural platelet count in the adjusted model. This finding should be interpreted cautiously. Portal vein diameter is only an indirect surrogate of portal hemodynamics and does not directly measure portal pressure, spleen volume, splenic sequestration, or thrombopoietic reserve (Yunusa et al., 2024). Although portal hypertension is commonly associated with thrombocytopenia through hypersplenism and splenic sequestration (Peck-Radosavljevic, 2017), the positive association observed in this cohort should not be interpreted as evidence that portal hypertension enhances the response to lusutrombopag. Instead, it may reflect residual confounding, selection of patients for procedures, timing of platelet measurements, or other unmeasured factors in real-world clinical practice.

### 4.6 Safety considerations

In this cohort, one thrombotic event was observed in the ALBI grades 1–2 group, and no thrombotic events were observed in the ALBI grade 3 group. Bleeding events were also observed only in the ALBI grades 1–2 group, including procedure-related bleeding after pulmonary ablation, transarterial lipiodol embolization, and hepatic resection, as well as one bleeding event in a patient who did not undergo an invasive procedure. No fatal bleeding or treatment-related death was observed. However, the absence of bleeding or thrombotic events in the ALBI grade 3 group should not be interpreted as evidence of equivalent or established safety, given the small number of ALBI grade 3 patients and the descriptive nature of the analysis.

Procedure heterogeneity further limits interpretation of bleeding risk. The cohort included procedures with different bleeding risk profiles, as well as patients who did not undergo an invasive procedure. Therefore, overall bleeding rates based on the full analysis set may not accurately reflect procedure-related bleeding risk. To address this issue, procedure-related bleeding was evaluated among patients who underwent invasive procedures, whereas bleeding events in patients without procedures were reported separately. Taken together, these safety findings are descriptive and hypothesis-generating. Larger prospective studies with standardized procedure categories, predefined bleeding definitions, systematic thrombosis surveillance, and sufficient representation of patients with poor hepatic reserve are needed to better characterize safety in this population.

### 4.7 Study limitations

This study has several limitations. First, it was a retrospective, single-center observational study with a limited sample size, particularly in the ALBI grade 3 subgroup. The study was therefore underpowered for definitive comparisons between ALBI strata, and non-significant findings should not be interpreted as evidence of equivalence or absence of effect. The possibility of type II error remains substantial. Because patients were selected for lusutrombopag treatment and planned procedures according to clinical judgment, selection bias and residual confounding cannot be excluded.

Second, treatment and procedure-related factors were not standardized. Although all patients received lusutrombopag at a fixed dose of 3 mg once daily, treatment duration, procedure timing, platelet measurement timing, intervention thresholds, use of rescue therapy, and procedure selection were determined by treating physicians in routine clinical practice rather than by a predefined protocol. These decisions were likely influenced by baseline platelet count, hepatic reserve, perceived bleeding risk, procedure type, procedural urgency, and the observed platelet response after treatment. As a result, patients with poorer hepatic reserve may have been selected for procedures only when their baseline platelet count or clinical condition was considered acceptable, whereas patients with better hepatic reserve may have proceeded to invasive procedures despite lower baseline platelet counts. Procedures may also have been delayed until platelet counts increased or supplemented by rescue therapy when platelet response was considered insufficient. Therefore, indication bias and selection bias may have attenuated, obscured, or otherwise distorted between-group differences in peri-procedural platelet counts and safety outcomes. The study also did not include pharmacokinetic or pharmacodynamic sampling; therefore, explanations involving altered protein binding or free drug exposure remain hypothesis-generating. Finally, safety outcomes were descriptive, with few bleeding or thrombotic events and no systematic imaging surveillance for portal vein thrombosis. Larger prospective multicenter studies with standardized treatment schedules, predefined platelet measurement timing, stratification by both ALBI grade and Child–Pugh class, and structured safety monitoring are needed to validate these findings.

## 5 Conclusion

In conclusion, this retrospective real-world study provides exploratory ALBI-stratified data on peri-procedural platelet outcomes after lusutrombopag treatment in HCC patients with thrombocytopenia. In exploratory analyses adjusted for baseline platelet count and portal vein diameter, no independent association between ALBI grade 3 and lower peri-procedural platelet count was detected in this selected cohort. This finding should not be interpreted as evidence of comparable platelet response, equivalent efficacy, or established safety across ALBI strata. Larger prospective studies with standardized treatment schedules, sampling time points, and structured safety surveillance are needed to validate these findings and to better define platelet response and safety in HCC patients with poorer hepatic reserve.

## Data Availability

The original contributions presented in the study are included in the article/supplementary material, further inquiries can be directed to the corresponding author.
